# Neuroprotective Effects of Fingolimod Supplement on the Retina and Optic Nerve in the Mouse Model of Experimental Autoimmune Encephalomyelitis

**DOI:** 10.3389/fnins.2021.663541

**Published:** 2021-04-26

**Authors:** Tao Yang, Zheng Zha, Xiao Yang, YueZhi Kang, Xin Wang, Yanping Tong, XueSong Zhao, Lei Wang, YongPing Fan

**Affiliations:** ^1^Beijing Tiantan Hospital, Capital Medical University, Beijing, China; ^2^School of Traditional Chinese Medicine, Capital Medical University, Beijing, China; ^3^School of Management Science and Engineering, Shandong University of Finance and Economics, Jinan, China; ^4^Dongzhimen Hospital, Beijing University of Chinese Medicine, Beijing, China; ^5^School of Traditional Chinese Medicine, Capital Medical University, Beijing, China

**Keywords:** multiple sclerosis, fingolimod, EAE, retinal degeneration, optic nerve, glial response

## Abstract

Favorable effects exerted by long-term administration of fingolimod therapy in multiple sclerosis (MS) patients have been reported, but sporadic side effects, such as reversible macular edema, also have been recorded. The present study aimed to determine whether fingolimod therapy is beneficial to the visual system in experimental autoimmune encephalomyelitis (EAE) mice. A decrease in demyelination and axon loss in the optic nerve as well as cellular infiltration, especially the recruited macrophages, was observed in EAE with fingolimod treatment. Fingolimod administration diminished hypergliosis of macroglia, including astrocytes and Müller cells in the retina and optic nerve in EAE. Microglia were hyperactivated in the retina and optic nerve in the EAE mice compared to controls, which could be alleviated by fingolimod treatment. Moreover, apoptosis of retinal ganglion cells (RGC) and oligodendrocytes in the optic nerve was significantly reduced with fingolimod treatment compared to that in the untreated EAE mice. These results suggested that fingolimod exerts neuroprotective and anti-inflammatory effects on the retina and optic nerve in a mouse model of EAE. Considering the paradox of favorable and side effects of fingolimod on visual system, we speculate that side effects including macular oedema caused by fingolimod during MS treatment is tendency to be vasogenic rather than hypergliosis in optic nerve and retina which warrants further neuroophthalmological investigation.

## Graphical Abstract

Our results show that fingolimod is a potential therapeutic approach to prevent EAE-induced visual impairment. Moreover, we show that the observed neuro-ophthalmological protective effects of fingolimod were dependent on hampering hyper-gliosis in the retina and optic nerve.

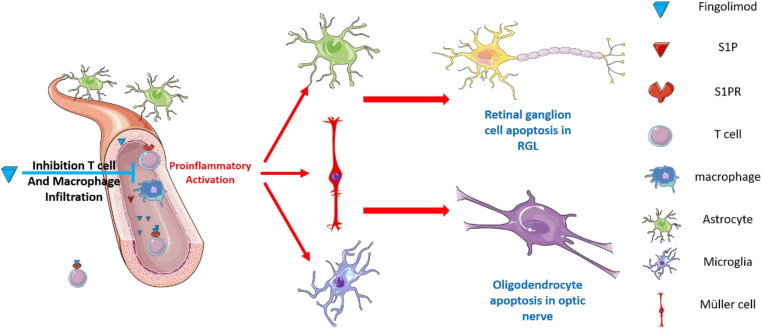

## Introduction

Multiple sclerosis (MS) is a chronic inflammatory and neurodegenerative disease and the most common non-traumatic cause of neurological disability in young adults, usually with a relapsing-remitting pattern (Karussis, 2014; Sospedra and Martin, 2016). Current immunomodulatory therapies for MS effectively inhibit autoimmune responses at the remitting stage, improving the outcomes by reducing the number of relapses while attenuating neurological disability (Derfuss et al., 2020). Fingolimod (FTY720), a structural analog of sphingosine 1-phosphate (S1P) receptor modular agent, is the first oral drug approved for the treatment of relapsing remitting multiple sclerosis (RRMS) (Derfuss et al., 2020). Pharmacologically, it acts as a selective antagonist of S1P1 subtype and a non-selective agonist of S1P receptors by downregulating receptor induction (Aktas et al., 2010; Huwiler and Zangemeister-Wittke, 2018). Fingolimod acts as an S1P modulator and prevents the egress of immune cells from secondary lymphoid tissues, depleting their abundance from the circulation; S1P is a crucial component in the regulation of lymphocyte trafficking (Tsai and Han, 2016; Huwiler and Zangemeister-Wittke, 2018). In phase II and III human MS trials, fingolimod showed promising therapeutic effects by decreasing the number of relapses and attenuating neurological disabilities (Cohen et al., 2010; Kappos et al., 2010; Chitnis et al., 2018). In MS and other demyelinating disease of the central nervous system (CNS), vision problems are the first sign of the disease in many cases, suggesting that glia and RGC in the optic nerve and retina are more sensitive to pathological conditions than cells in other parts of the CNS. However, the effects of fingolimod on the visual system, often affected by RRMS, are not yet fully known.

Favorable effects exerted by long-term administration of fingolimod therapy in MS patients have been reported, but a few sporadic unwanted effects, such as reversible macular edema, also have been documented (Gelfand et al., 2012; Zarbin et al., 2013; Mandal et al., 2017). Considering that the current anecdotal reports of fingolimod associated macular edema (FAME) and other retinal complications, coupled with the paucity of information about the putative pathogenic mechanisms responsible for the impact of fingolimod on the visual system, we utilize a MS animal model which lead to a prevalence of RGC loss and optic neuritis to determine whether fingolimod therapy is beneficial to the visual system in experimental autoimmune encephalomyelitis (EAE). Since glial cells possess abundantly S1PR and share a large proportion in the optic nerve and retina, and they are critical important for maintenance of neuronal activity in the CNS, especially the visual system, the effects of fingolimod on retinal glial (microglial, astrocyte, and Müller cells) and optic nerve glial (microglial and macroglial) responses have been investigated.

## Materials and Methods

### Animals

A total of 48 C57BL/6 female mice of specific pathogen-free (SPF) grade (age, 4–6 weeks; weight, 18–20 g) were obtained from Beijing Vital River Experimental Animal Tech. Co., Ltd., China and housed at the Center of Laboratory Animals at Capital Medical University [certification no. SYXK (JING) 2010-0020]. A group of five mice was housed in individual cages at 18–25°C and humidity (55 ± 10%)-controlled room with 12-h light/dark cycle. The cages were cleaned every 3 days. The study was approved by the Ethics Committee of Capital Medical University (AEEI-2019-186).

### EAE Induction

Mice were randomly divided into four groups: the normal group (*n* = 12), one untreated EAE group (*n* = 12), and two EAE groups that were administered 0.3 mg/kg (*n* = 12) and 1 mg/kg (*n* = 12) of fingolimod (Basel, Switzerland), respectively. EAE was induced by injecting 0.2 mL emulsion containing 50 μg MOG_35__–__55_ peptide (Beijing SciLight Biotechnology Co., Ltd., Beijing, China) subcutaneously in 100 μL of normal saline (NS) and 300 μg *Mycobacterium tuberculosis* (Sigma Aldrich, St. Louis, MO, United States) in 100 μL of complete Freund’s adjuvant (CFA) (Sigma Aldrich). Additionally, the immunized mice received 200 ng of pertussis toxin (PTX) (Sigma Aldrich) on day 0 and 2 post-immunization (PI). The non-immunized control mice group received NS without MOG_35__–__55_ peptide, PTX, and CFA. To evaluate the effects of fingolimod, the EAE mice were treated with 0.3 and 1 mg/kg fingolimod solution orally in 0.2 mL of H_2_O from day 12 when 50% of the immunized animals developed clinical signs of EAE. However, the non-immunized control and EAE groups of mice received 0.2 mL of H_2_O on day 12.

### Clinical Assessment

The neurological function scores of the EAE mice were assessed daily using a five-point scoring system; 0 = no clinical signs (normal mouse), 0.5 = partial loss of tail tonus (normal gait), 1 = paralyzed tail (normal gait), 2 = moderate hind limb paraparesis (hind limb weakness), 2.5 = severe hind limb paraparesis, 3 = partial hind limb paralysis (inability to right itself within 5 s after being placed on the back), 3.5 = hind limb paralysis (dragging both hind limbs), 4 = hind limb paralysis plus partial front leg paralysis, 4.5 = tetraplegia, moribund (no movement), and 5 = death (Giralt et al., 2013).

### Mouse Eyeball and Optic Nerve Preparation

The mice were sacrificed by injecting sodium pentobarbital (100 mg/kg body weight) on day 35 after immunization. The optic nerves were dissected and immersed into 4% paraformaldehyde immediately. The eyeball of each mouse was pierced from the ocular angle before immersion into 4% paraformaldehyde.

### Determination of Myelin and Axon Degeneration of the Optic Nerve by Silver Staining

The deparaffinized samples were transferred into distilled water; fixed in 4% formaldehyde solution for 5 min at room temperature, and immersed in cupric glycinate at 37°C for 5 min. Subsequently, the slides were slanted to allow the silver solution runoff and immersed in a solution (pyrogallol, 1 g; distilled water, 45 mL; dehydrated alcohol, 55 mL; 1% nitric acid solution, 0.2 mL) for 1 min at 45°C, washed in 50% alcohol for 5 s, followed by treatment in a toning bath solution (auri chloridum 1 g; distilled water 200 mL; glacial acetic acid 0.2 mL) for a few minutes until the light yellow-brown silver tint faded away/Subsequently, the slides were washed and immersed into solution (50% alcohol 100 mL; aniline oil 2 drops) for 15 s, washed and immersed into a solution (sodium thiosulfate 5 g added distilled water to 100 mL) for 1 min, washed, and sealed with neutral balsam (Palmgren, 1948).

### Determination of Inflammatory Infiltration of the Retina and Optic Nerve by Hematoxylin and Eosin

Paraffin sections of the optic nerve and retina were stained with Hematoxylin and Eosin (H&E), luxol fast blue staining (LFB), and silver. Five images of each optic nerve (anterior, medial, and posterior) and retina sections were taken under a fluorescence microscope (Olympus, Tokyo, Japan) at ×400 magnification. The inflammatory cell infiltration was established using a four-point score on H&E staining: 0 = no infiltrate; 1 = scattered inflammatory infiltration; 2 = moderate inflammatory infiltration; 3 = severe inflammatory infiltration; 4 = massive inflammatory infiltration.

### Immunofluorescence Analysis of the Retina and Optic Nerve

Paraffin slices were incubated in primary antibodies: GFAP (#12389, CST, Boston, United States), vimentin (#5741, CST), Iba1 (#17198, CST), Brn3a (ab245230, Abcam, Cambridge, United Kingdom), CC1 (MABN50, Millipore, Massachusetts, United States) overnight at 4°C. The slices were washed three times with phosphate-buffered saline (PBS) and incubated with corresponding secondary antibodies at 37°C for 60 min and then washed three times with PBS. Then, parts of paraffin slices (co-staining of Iba1/TMEM119, Brn3a/cleaved caspase 3, CC1/cleaved caspase 3) were incubated in primary antibodies: cleaved caspase 3 (#9661, CST) and TMEM119 (ab209064, Abcam) overnight at 4°C. The slices were washed three times with PBS and incubated with corresponding secondary antibodies at 37°C for 60 min, and the nucleus was co-stained with DAPI at 4°C within 3 days for image capturing.

### Reverse Transcription-Polymerase Chain Reaction

Total RNA was extracted using TRIzol (Invitrogen, United States) and digested by DNase I for eliminating DNA fragments. Amplification was performed on a Roche LightCycler^®^ 480II according to the manufacturer’s instructions. The cycle threshold (2^–ΔΔCt^) method was used to calculate the levels of RNA expression. Specific primer pairs for b*-actin*: 5′-GAGATTACTGCTCTGGCTCCTA-3′ and 3′–GGA CTCATCGTACTCCTGCTTG-5′; *Caspase3*: 5′-GTGAAGAGT TGGACCACCATAGCA-3′ and 3′-AATGAGTCAACCAAGT TCGCACAC-5′; *Iba1*: 5′-TCCGAGGAGACGTTCAGCTACTC-3′ and 3′-TCTGACTCTGGCTCACGACTGTT-5′; *GFAP*: 5′-GA ACAACCTGGCTGCGTATAGACA-3′ and 3′-CTGCCTCGTA TTGAGTGCGAATCT-5′; *brn3a*: 5′-TCGCTCACGCTCTCG CACAA-3′ and 3′-CTTCTTCTCGCCGCCGTTGAA-5′; V*imentin*: 5′-GTCCACACGCACCTACAGTCTG-3′ and 3′-CGAGAAGTCCACCGAGTCTTGAAG-5′.

### Image Analysis

Fluorescence micrographs were acquired using Pannoramic Scan (Hungary). To analyze the images for counting the number of cells, each image labeled with random numbers was analyzed by Image J software (NIH). Cells stained with Iba1, Brn3a, TMEM119, and CC1 were calculated in each image by particle analysis. The percentage of apoptotic mature oligodendrocytes (CC1+) and RGC (Brn3a+) was calculated by co-staining cleaved caspase 3. The number of resident microglia (Iba1+) was calculated based on TMEM119-positive cells, and the number of circulating macrophages (Iba1+) was calculated by subtracting the number of TMEM119-positive cells. To determine the fluorescence and integral optical density (IOD), each image labeled with GFAP, vimentin, and LFB was analyzed by signal area using Image J according to the manufacturer’s guidelines.

### Statistical Analysis

Statistical analysis was carried out using GraphPad Prism version 8 (San Diego, CA, United States). The clinical performance scores were reported as mean ± standard deviation (SD), and the figure measurement data were reported as mean ± standard deviation (SD). Normal distribution and equal variances were examined in all groups. Multigroup comparisons and *post hoc* contrasts were carried out by one-way analysis of variance (ANOVA) test and Tukey’s multiple comparison test, respectively. *P* < 0.05 indicated statistical significance.

## Results

### Lower Neurological Disability in Mice Receiving Fingolimod

**FIGURE 1:**
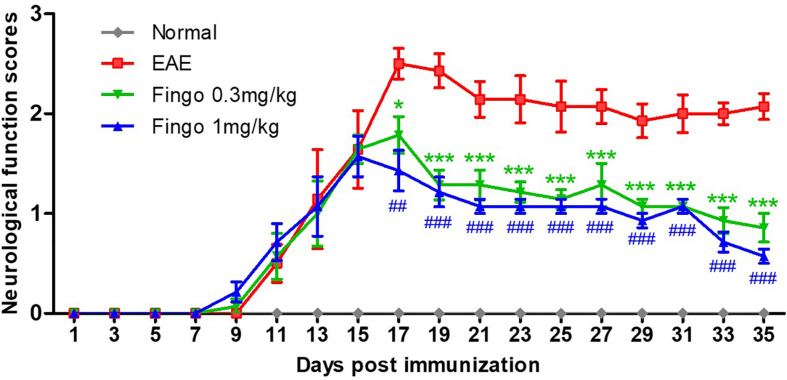
Clinical function scores of EAE mice. Scores are presented as mean ± SD. The treated group was given fingolimod at the dose of 0.3 and 1 mg/kg, respectively, from day 12 when 50% of immunized animals had developed clinical signs of EAE. Values represent mean ± SD. One-way ANOVA plus Turkey’s multiple comparisons. Compared to the control group: *P < 0.05, ***P < 0.001, ^##^*P* < 0.01, ^###^*P* < 0.001.

Immunized mice developed neurological signs starting from day 8, and all immunized mice developed clinical signs of EAE. The average neurological score of the EAE mice rose to the peak on day 17 (2.50 ± 0.15), followed by a decrease due to partial remission at days 19–21. A relatively stable phase was formed from days 21 to 35. The EAE mice administered fingolimod (0.3 and 1 mg/kg) showed less neurological disability compared to the EAE model. The scores of the 0.3 and 1 mg/kg fingolimod groups decreased from days 17 to 35. From day 17, the EAE scores for the fingolimod treatment groups were significantly lower than those for the untreated group (Figure 1).

### Less Inflammatory Cell Infiltration in the Optic Nerve With Fingolimod Treatment

**FIGURE 2:**
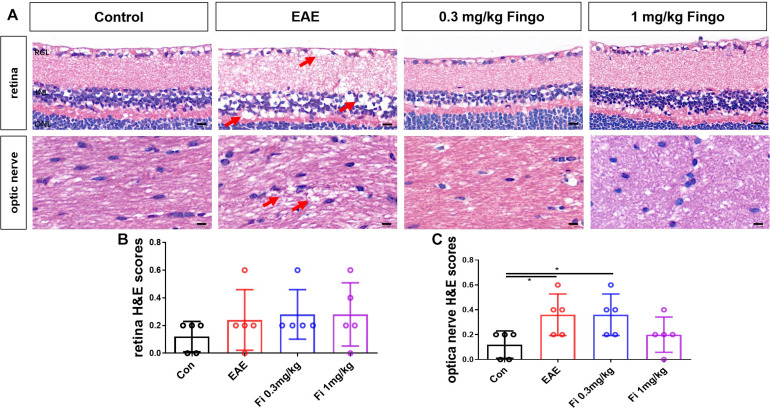
Inflammatory cell infiltration in the optic nerve with fingolimod treatment. **(A)** Retina and optic nerve were stained with H&E. The degeneration of the retina and optic nerve was indicated by arrows. **(B)** Cellular infiltration was measured by H&E scores in the retina. **(C)** The cellular infiltration was measured by H&E scores in the optic nerve. Values represented as mean ± SD. One-way ANOVA plus Tukey’s multiple comparisons. *P < 0.05, **P < 0.01, ***P < 0.001. Scale bar = 5 μm.

To evaluate the effects of fingolimod on the optic nerve and retina, sections were stained with H&E and scored. The H&E scores revealed a large number of inflammatory cells in the optic nerve of EAE mice, whereas scattered inflammatory cells were noted in unimmunized mice on day 35 postimmunization (PI). The present data showed that treatment with fingolimod (1 mg/kg) alleviates the infiltration of inflammatory cells in the optic nerve (*P* = 0.49) (Figure 2C). The H&E showed strong structural degeneration in the retina and optic nerve (arrows, Figures 2A,B), whereas it could not be fully explained by moderate inflammatory infiltration. This phenomenon indicated that the resident immune cells might be involved in the degeneration of the optic nerve and retina.

### Less Demyelination and Axon Degeneration in the Optic Nerve With Fingolimod Treatment

**FIGURE 3:**
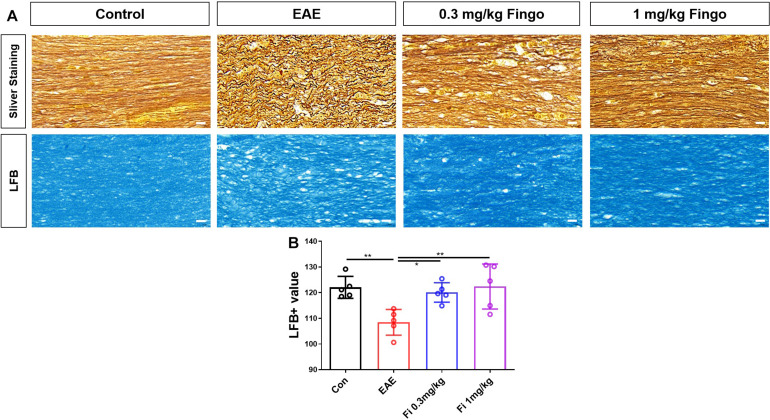
Demyelination and axon degeneration in the optic nerve with fingolimod treatment. **(A)** Axons and myelin in the optic nerve were marked with sliver staining and LFB. **(B)** Demyelination analyzed by LFB scores in the optic nerve (mean gray value). Values are presented as mean ± SD. One-way ANOVA plus Turkey’s multiple comparisons. *P < 0.05, **P < 0.01, ***P < 0.001. Scale bar = 10 μm.

Based on the H&E results, myelin and axons of RGC in the optic nerve were detected by silver staining and LFB. Consistent with previous findings, the axons were injured and tangled in the optic nerve in EAE mice; however, this phenomenon was reduced in the fingolimod-treated group (Figure 3A). In addition to axon degeneration, demyelination also occurred in the optic nerve (Figure 3B). The mice with EAE had less LFB mean value compared to normal mice, whereas the immunized mice with fingolimod treatment showed higher LFB, indicating demyelination [Con vs. EAE (*P* = 0.009), EAE vs. Fi 0.3 mg/kg (*P* = 0.027), EAE vs. Fi 1 mg/kg (*P* = 0.008)] (Figure 3C).

### Less Oligodendrocyte Apoptosis in the Optic Nerve With Fingolimod Treatment

**FIGURE 4:**
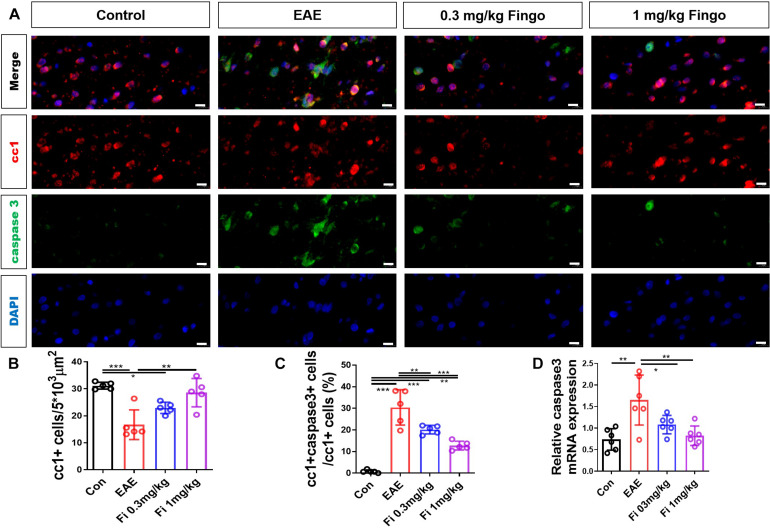
Oligodendrocyte apoptosis with fingolimod treatment. **(A)** Oligodendrocytes and apoptotic cells in the optic nerve were marked with cc1 and cleaved caspase 3; the apoptotic oligodendrocytes were labeled both. **(B)** Number of cc1+ cells in the optic nerve (area = 5 × 10^3^ μm^2^). **(C)** Ratios of cc1+ cleaved caspase 3+ to cc1+ cells in the optic nerve. **(D)** *Caspase 3* mRNA expression in optic nerve. Values are represented as mean ± SD. One-way ANOVA plus Tukey’s multiple comparisons. *P < 0.05, **P < 0.01, ***P < 0.001. Scale bar = 10 μm.

Considering the strong degeneration and moderate cell infiltration in the retina and optic nerve, we first investigated the apoptosis of retinal RGC and oligodendrocytes in the optic nerve. Next, we used a cc1 antibody to mark the oligodendrocyte. The apoptotic cells were stained with cleaved caspase three antibody. Compared to the control group, significantly fewer oligodendrocytes were detected in the optic nerve in the EAE mice [Con vs. EAE (*P* = 0.023)] (Figures 4A,B). Compared to the EAE group, higher numbers of oligodendrocytes were measured in the optic nerve with fingolimod treatment [EAE vs. Fi 1 mg/kg (*P* = 0.037)] (Figures 4A,B). The apoptotic oligodendrocytes were co-stained with cc1 and cleaved caspase 3 antibodies, and the ratios of cc1+ cleaved caspase 3+ to cc1+ cells were measured. Compared to the control group, significantly higher ratios of cc1+ cleaved caspase 3+ to cc1+ cells in the optic nerve with EAE indicated a higher apoptotic oligodendrocyte in the immunized mice [Con vs. EAE (*P* < 0.001)] (Figures 4A,C). Under fingolimod treatment (0.3 and 1 mg/kg), the optic nerve of the EAE showed less apoptotic ratio and more preserved oligodendrocytes compared to the untreated EAE mice [EAE vs. Fi 0.3 mg/kg (*P* = 0.009); EAE vs. Fi 1 mg/kg (*P* < 0.001)] (Figures 4A,C).

RT-PCR assays on total RNA from optic nerve samples showed that the expression of *Caspase 3* gene was downregulated by fingolimod treatment compared to control mice [Con vs. EAE (*P* = 0.001); EAE vs. Fi 0.3 mg/kg (*P* = 0.049); EAE vs. Fi 1 mg/kg (*n* = 5; *P* = 0.003)] (Figure 4D).

### Less Retina Ganglion Cell Apoptosis in the Retina With Fingolimod Treatment

**FIGURE 5:**
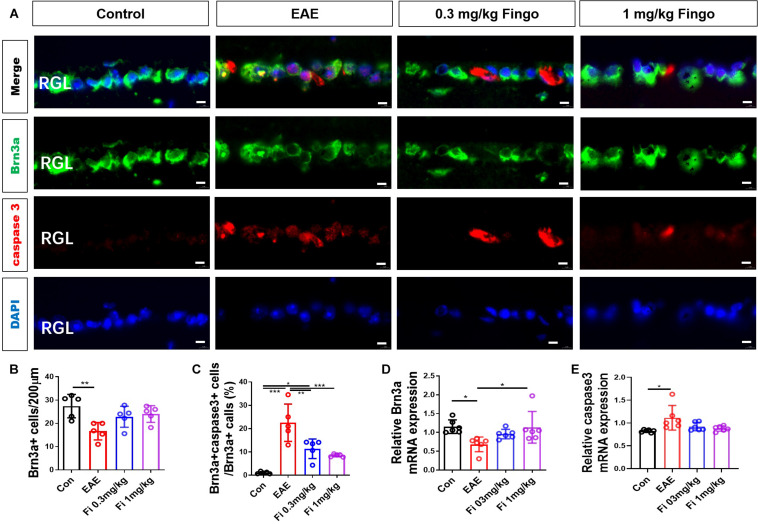
Retina ganglion cell apoptosis with fingolimod treatment. **(A)** Retinal ganglion cells and apoptotic cells in the RGC were marked with Brn3a and cleaved caspase3; the apoptotic retinal ganglion cells were labeled with both antibodies. **(B)** Number of Brn3a+ cells in the RGC (length = 200 μm). **(C)** Ratios of Brn3a+ cleaved caspase3+ to Brn3a+ cells in the optic nerve. **(D–E)** *Brn3a* and *Caspase 3* mRNA expression in the retina. Values are presented mean ± SD. One-way ANOVA plus Tukey’s multiple comparison. *P < 0.05, **P < 0.01, ***P < 0.001. Scale bar = 5 μm.

On the retina, we used a Brn3a antibody to mark the RGCs. The apoptotic cells were stained with cleaved caspase 3 antibody. Similar to the optic nerve, significantly fewer ganglion cells were detected in the retinal ganglion layer (RGL) in the EAE mice compared to the control group [Con vs. EAE (*P* = 0.004)] (Figures 5A,B). Compared to the untreated EAE group, higher numbers of RGCs were preserved in the RGL with fingolimod treatment [EAE vs. Fi 1 mg/kg (*P* = 0.044)] (Figures 5A,B). The apoptotic RGCs were co-stained with Brn3a and cleaved caspase 3 antibodies, and the ratio of Brn3a+ cleaved caspase 3+ to Brn3a+ cells was measured. Compared to the control group, significantly higher ratios of Brn3a+ cleaved caspase 3+ to Brn3a+ cells were observed in the RGLs with EAE, indicating high apoptotic RGCs in immunized mice [Con vs. EAE (*P* < 0.001] (Figures 5A,C). Under fingolimod treatment, the RGCs of EAE showed less apoptotic ratio and more preserved RGCs compared to the untreated EAE mice [EAE vs. Fi 0.3 mg/kg (*P* = 0.006); EAE vs. Fi 1 mg/kg (*P* = 0.001)] (Figures 5A,C).

RT-PCR assays on total RNA from retina samples showed that *Brn 3a* gene expression was upregulated by fingolimod treatment compared to the EAE mice [Con vs. EAE (*P* = 0.022); EAE vs. Fi 1 mg/kg (*P* = 0.029)] (Figure 5D) and the *Caspase 3* gene expression was upregulated by immunization [Con vs. EAE (*P* = 0.013)] (Figure 5E).

### Less Gliosis of Müller Cells in the Retina With Fingolimod Treatment

Considering the severity of retinal degeneration and moderate inflammatory cell infiltration, we postulated that local macroglia (astrocytes and Müller cells) might be involved in the degeneration of the optic nerve and retina. Gliosis occurs when Müller cells and astrocytes respond to the retina and optic nerve insults, resulting in a series of morphological and molecular changes (Brambilla, 2019). Proinflammatory mediators released by surrounding neurons, microglia, and other cells in response to (CNS) injury cause changes associated with astrocytes and Müller cell reactivation (Liddelow et al., 2017). The primary morphological change during reactive astrocytes and Müller cell gliosis is the hypertrophy of processes, which is linked to increased expression of intermediate filaments, especially GFAP and vimentin. We used GFAP and vimentin antibodies to mark retina astrocytes and Müller cells.

**FIGURE 6:**
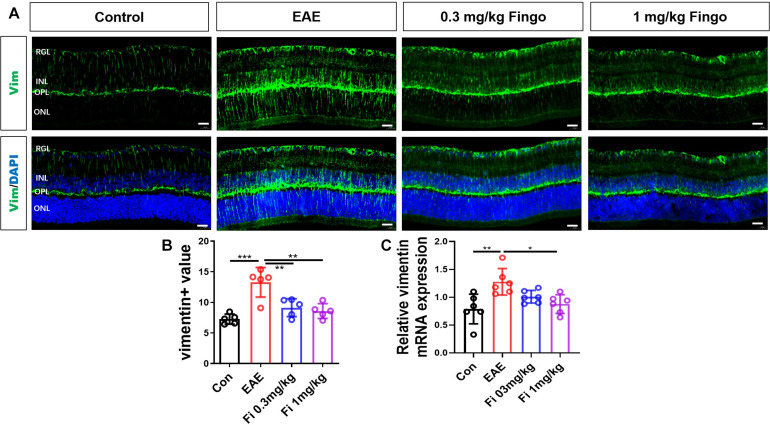
Morphological and mean gray value changes of vimentin + cells. **(A)** Vimentin antibody was used to label the Müller cells in the retina. Vimentin + cells had a longer process, which extended from OPL to deeper ONL and RGL in the EAE group. **(B)** Comparison of Vimentin mean gray values among groups. **(C)** *Vimentin* mRNA expression in the retina. RGL, retinal ganglion layer; INL, inner nuclear layer; OPL, outer plexiform layer; ONL, outer nuclear layer. Values represent mean ± SD. One-way ANOVA plus Tukey’s multiple comparisons. *P < 0.05, **P < 0.01, ***P < 0.001. Scale bar = 20 μm.

Compared to the control group, vimentin + cells had longer processes, which extended from OPL into the deeper outer nuclear layer (ONL) and RGL [Con vs. EAE (*P* < 0.001] (Figures 6A,B). Treatment with 0.3 and 1 mg/kg fingolimod significantly lowered the expression of vimentin compared to that in the EAE group [EAE vs. Fi 0.3 mg/kg (*P* = 0.004); EAE *vs*. Fi 1 mg/kg (*P* = 0.001)] (Figures 6A,B). Compared to the 0.3 mg/kg fingolimod-treated group, the group with 1 mg/kg showed a decreased expression of vimentin in the entire depth of the retina [Fi 0.3 mg/kg vs. Fi 1 mg/kg (*P* = 0.001)] (Figures 6A,B).

RT-PCR assays on total RNA from retina samples showed that the expression of *Vimentin* gene was downregulated by fingolimod treatment compared to EAE mice [Con vs. EAE (= 0.003); EAE vs. Fi 1 mg/kg (*P* = 0.015)] (Figure 6C).

### Less Gliosis of Astrocytes in the Retina and Optic Nerve With Fingolimod Treatment

**FIGURE 7:**
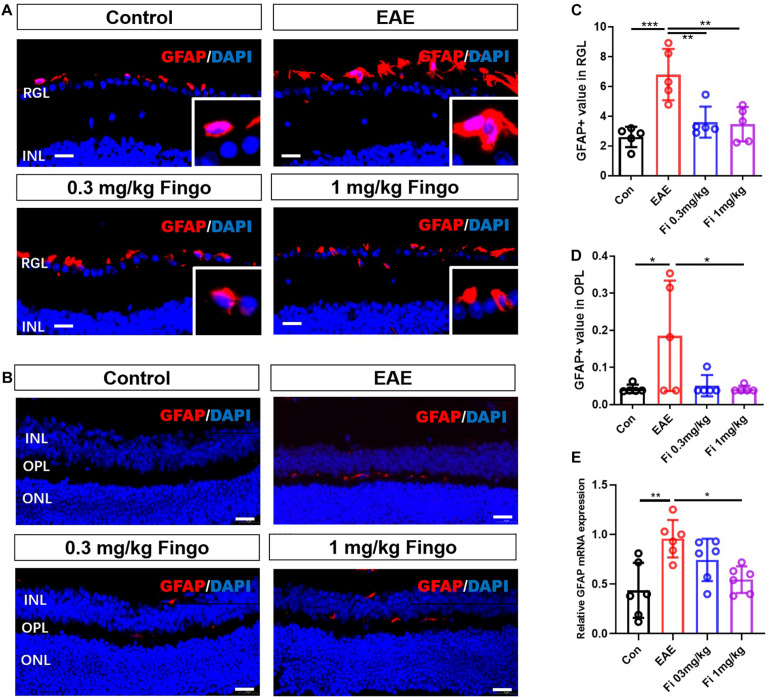
Morphological and mean gray value changes of the GFAP+ cells in the retina. **(A)** GFAP antibody was used to label astrocytes in the retina. GFAP+ cells were primarily located in the RGL, and the cell body was hypertrophied in the EAE mice. **(B)** Comparisons of the GFAP mean gray value among groups in RGL. **(C)**. GFAP+ cells were slightly located in the OPL. **(D)** Comparisons of the GFAP mean gray value among the groups in OPL. **(E)** *GFAP* mRNA expression in retina. RGL, retinal ganglion layer; INL, inner nuclear layer; OPL, outer plexiform layer; ONL, outer nuclear layer. Values are represented mean ± SD. One-way ANOVA plus Tukey’s multiple comparisons. *P < 0.05, **P < 0.01. Scale bar = 20 μm.

**FIGURE 8:**
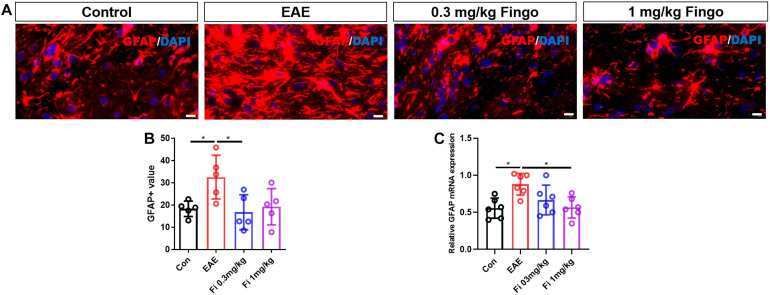
Morphological and mean gray value changes of GFAP+ cells in the optic nerve. **(A)** Astrocytes labeled with GFAP with hypertrophy in EAE mice. **(B)** Comparison of the GFAP mean gray value among groups in the optic nerve. **(C)** *GFAP* mRNA expression in retina. Values are presented as mean ± SD. One-way ANOVA plus Tukey’s multiple comparisons. *P < 0.05, **P < 0.01. Scale bar = 10 μm.

Compared to the control group, significant gliosis was observed in the optic nerve of EAE mice. The administration of fingolimod significantly diminished the GFAP expression and could be detected compared to the EAE group in RGL (*P* < 0.001) (Figures 7A,B), OPL (*P* = 0.022), and optic nerve (*P* = 0.018) (Figures 7A–D, 8A,B). No significant difference could be observed between the 0.3 and 1 mg/kg fingolimod groups in the optic nerve [Fi 0.3 mg/kg vs. Fi 1 mg/kg (*P* = 0.956)] (Figures 8A,B). Previous studies identified difficulties in the measurement of astrocytes in the optic nerve, which might explain the lack of significant statistical difference between EAE and fingolimod (1 mg/kg)-treated groups in the optic nerve.

RT-PCR assays on total RNA from retina and optic nerve samples showed that *GFAP* gene expression was downregulated by fingolimod treatment compared to EAE mice in the retina [Con vs. EAE (*P* = 0.002); EAE vs. Fi 1 mg/kg (*P* = 0.014)] (Figure 7E) and optic nerve [Con vs. EAE (*P* = 0.011); EAE vs. Fi 1 mg/kg (*P* = 0.014)] (Figure 8C).

### Less Microglia and Recruited Phagocytes in the Retina and Optic Nerve With Fingolimod Treatment

**FIGURE 9:**
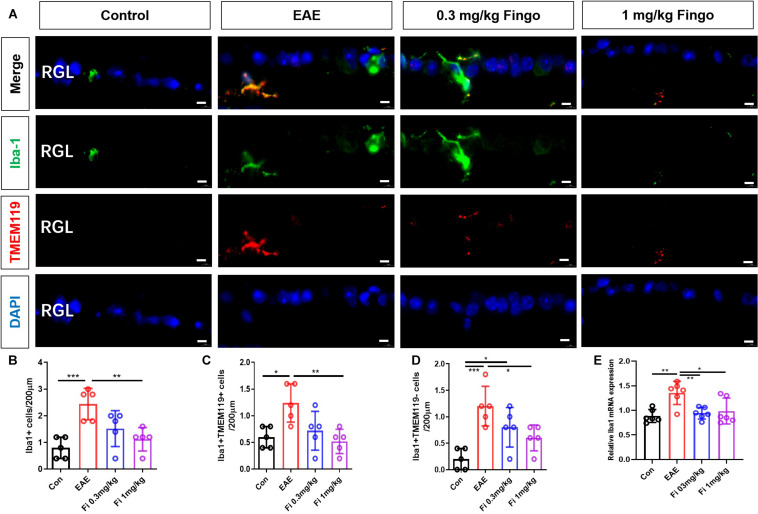
Resident microglia and recruited macrophage numbers in the retina with fingolimod treatment. **(A)** Resident microglia and recruited macrophages were labeled with Iba1+ TMEM119+ and Iba1+ TMEM119- in the retina, respectively. **(B)** Comparison of microglia and macrophage numbers among groups in RGL (length = 200 μm). **(C)** Comparison of microglia numbers among groups in RGL (length = 200 μm). **(D)** Comparison of macrophage numbers among groups in RGL (length = 200 μm). **(E)** *Iba1* mRNA expression in retina RGL, retinal ganglion layer. Values are indicated as mean ± SD. One-way ANOVA plus Tukey’s multiple comparisons. *P < 0.05, **P < 0.01, ***P < 0.001. Scale bar = 5 μm.

**FIGURE 10:**
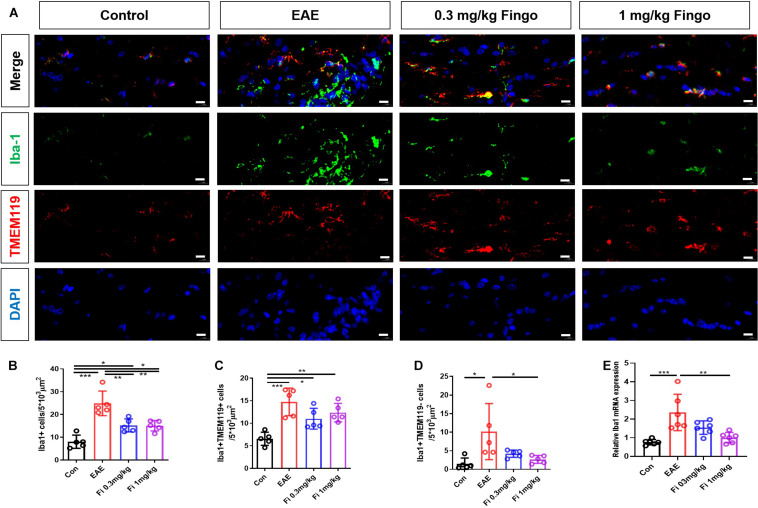
Resident microglia and recruited macrophage numbers in the optic nerve with fingolimod treatment. **(A)** Resident microglia and recruited macrophages were labeled with Iba1+ TMEM119+ and Iba1+ TMEM119- in the optic nerve, respectively. **(B)** Comparison of microglia and macrophage numbers among groups in the optic nerve (area = 5 × 10^3^ μm^2^). **(C)** Comparison of microglia numbers among groups in RGL (area = 5 × 10^3^ μm^2^). **(D)** Comparison of macrophage numbers among groups in RGL (area = 5 × 10^3^ μm^2^). **(E)** *Iba1* mRNA expression in retina. Values are mean ± SD. One-way ANOVA plus Tukey’s multiple comparisons. *P < 0.05, **P < 0.01, ***P < 0.001. Scale bar = 5 μm.

Microglia are the resident macrophages during CNS maturation. Considering that the proinflammatory resident microglia and recruited macrophages might be involved in the degeneration of the retina and optical nerve, we also labeled microglia (Iba1+ TMEM119+) and macrophages (Iba1+ TMEM119−) in the retina and optic nerve. Compared to the control group, a significant increase was noted in the total number of microglia and macrophages in the retina [Con vs. EAE (*P* = 0.001)] (Figures 9A,B) and optic nerve [Con vs. EAE (*P* < 0.001] (Figures 10A,B) in EAE mice. Under the administration of fingolimod (0.3 and 1 mg/kg), a significantly diminished number of microglia and macrophages were detected in RGL [EAE vs. Fi 1 mg/kg (*P* = 0.006)] and optic nerve compared to those in the untreated EAE group [EAE vs. Fi 0.3 mg/kg (*P* = 0.003); EAE vs. Fi 1 mg/kg (*P* = 0.002)] (Figures 9A–D, 10A–D). Although it seemed that fingolimod treatment (0.3 mg/kg) reduces the number of microglia and macrophages in RGL, no significant statistical difference was detected between the EAE and fingolimod-treated groups [EAE vs. Fi 0.3 mg/kg (*P* = 0.067)] (Figures 9A,B).

RT-PCR assays on total RNA from retina and optic nerve samples showed that the expression of *Iba1* gene was downregulated by fingolimod treatment compared to that in EAE mice retina [Con vs. EAE (*P* = 0.003); EAE vs. Fi 0.3 mg/kg (*P* = 0.009); EAE vs. Fi 1 mg/kg (*P* = 0.020)] (Figure 9E) and optic nerve [Con vs. EAE (*P* < 0.001; EAE vs. Fi 1 mg/kg (*P* = 0.002)] (Figure 10E).

## Discussion

The optic nerve is a specialized sensory nerve that’s responsible for vision. The axons of the optic nerve originate from the RGC and end at the lateral geniculate body after passing through the optic chiasm. The fiber sheath covering the optic nerve is the direct continuation of the three- meningeal layer (dura, arachnoid and pia mater), so the optic nerve is considered part of the brain, rather than the true cranial nerve. Importantly, due to this special anatomical structure of the optic nerve, it becomes susceptible to CNS and ocular diseases. Currently, there is no reliable treatment for optic neuritis, which often contributes to permanent visual disability. It is also the most common initial symptom of MS. Optic nerve degeneration, RGC loss, and local inflammatory cell activation are the characteristic symptoms of optic neuritis (Chan, 2002; Kolappan et al., 2009; Gonçalves et al., 2019). The neuroprotective effects of fingolimod in EAE have been reported. These include promoting clinical performance, hindering inflammation, and alleviating pathological injury (Rossi et al., 2012; Colombo et al., 2014; Qiu et al., 2018). In EAE, microglia and T cell influx might generate inflammatory lesions linked to demyelination and axonal degeneration in the CNS (Yamasaki et al., 2014; Chu et al., 2018; Zhang et al., 2018). Previous studies have focused on hyperglial activation as T cell-linked autoimmune responses that have been previously evaluated under fingolimod treatment (Kataoka et al., 2005; Bravo et al., 2016; Eken et al., 2017). The present study revealed another major aspect of fingolimod protective effects, especially in attenuating gliosis in the visual system. We evaluated the neuroprotective effects of fingolimod using a well-established animal model of MS induced by MOG_35__–__55_ peptide in C57BL/6 mice. Iba1, GFAP, TMEM119, and Vimentin were used as glia indicators, which suggested that the protective effects are the results of fingolimod acting on microglia, recruited macrophages, astrocytes, and Müller cells, hampering hyper-gliosis.

Microglia, the resident macrophages in the CNS, monitor the CNS and remove cellular detritus, which triggers the inflammatory processes in EAE development (Chu et al., 2018; Rothhammer et al., 2018). Blood-derived macrophages and microglia play a detrimental role in driving neuronal and myelin degeneration, including RGCs and oligodendrocytes in EAE. This phenomenon could be induced by ablating microglia populations or inhibiting microglia, resulting in delayed retina degeneration (Ponomarev et al., 2011; Horstmann et al., 2016). In the current study, we differentiated between recruited macrophages and resident microglia by TMEM119, which was not expressed by infiltrated macrophages. The results showed that the number of macrophages (Iba1+/TMEM119−) and microglia (Iba1+/TMEM119+) were markedly reduced in the EAE mice with fingolimod treatment in the retina and optic nerve. This corroborated with the findings from other studies on the brain and spinal cord of EAE mice, wherein fingolimod administration was associated with a decline in the number of microglia/macrophages. Thus, a therapeutic mechanism of fingolimod treatment on EAE could be effectuated by a decrease in microglia/macrophage response in the retina and optic nerve.

Astrocytes and Müller cells are the two major macroglia cells in the retina that constitute the blood-brain barrier and modulate immune molecules in the physiological and pathological states (Brambilla, 2019). Both types provide structural and trophic support to the surrounding cells. The primary morphological change during reactive astrocytes and Müller cell gliosis is the hypertrophy of processes linked to increased expression of intermediate filaments, especially GFAP and Vimentin (Hu et al., 2017; Lindenau et al., 2019). The initial responses of astrocytes and Müller cells in EAE are favorable and generate growth factors to promote recovery. However, chronic gliosis of astrocytes, and Müller cells impede recovery due to the release of cytotoxic factors (Rothhammer et al., 2017; Chao et al., 2019; Tassoni et al., 2019; Wheeler et al., 2019). Previous studies demonstrated that reactive astrocytes and Müller cells induced by microglia lose normal function and become neurotoxic, rapidly killing the neurons and mature differentiated oligodendrocytes (Liddelow et al., 2017; Rothhammer et al., 2018). Like astrocytes in the brain, Müller cells exhibited activated and reactive glial cells under pathological conditions. Typical features of gliosis in Müller cells include cell hypertrophy, upregulated expression of intermediate filaments, increased proliferation rate, and down-regulated expression of glutaminase synthetase (GS) (Bringmann et al., 2006). It was found that Müller cells co-cultured with activated microglia significantly increased the mRNA and protein expressions of pro-inflammatory factors such as IL-1β, IL-6, and iNOS (Wang and Wong, 2014). These pathological function of Müller cells can aggravate the inflammatory damage to RGC cells in EAE. So, targeting microglia activity, microglia-induced astrocyte and Müller cells gliosis alters the course of neural degeneration in the retina and optic nerve (Rothhammer et al., 2017; Groves et al., 2018). In the present study, results showed that hypergliosis of the astrocytes around the RGL layer and hypertrophic Müller cells span the depth of the retina in EAE could be significantly alleviated by fingolimod treatment. We also observed that neural degeneration in optic nerve in EAE could be benefited from the administration of fingolimod, thereby indicating that fingolimod inhibited astrocyte and Müller cell hypergliosis to protect the retinal cells and optic nerve from chronic inflammatory responses. The current results also showed that fingolimod supplements promote RGC and oligodendrocyte preservation in the MOG-induced EAE model, which could be attributed to its counteraction on microglia/macrophages and macroglia activation in the retina and optic nerve.

Given 0 of 425 RRMS patients receiving 0.5 mg fingolimod developed macular oedema and 7 out of 429 RRMS patients receiving 1.25 mg developed macular oedema in the FREEDOMS study, we have reviewed the relevant published literature with the aim of selecting appreciate fingolimod dosages (0.3 and 1 mg/kg) to represent low and high dosage of fingolimod treatment in MS patients (Chiba et al., 2011; Kappos et al., 2015; Zhang et al., 2015; Ambrosius et al., 2017). In the present study, we did not find direct evidence that high dosage of fingolimod treatment had negative effects on optic nerve and retina in EAE which could lead to macular oedema. And current results turn out that high dosage of fingolimod treatment has stronger neuroprotective effect on visual system in EAE. Considering this paradox, we speculate that macular oedema caused by fingolimod during MS treatment is tendency to be vasogenic rather than hypergliosis in optic nerve and retina as S1PRs have been found abundantly expressed on vascular endothelial and smooth muscle cells, and regulate their functions.

A limitation of the present study is that the neuroprotective effect of fingolimod cannot be ruled out the attribution of reduced immune cell infiltration. Another limitation is we did not perform Western blot as the total protein extracted from the retina and the optic nerve was limited.

## Conclusion

In conclusion, the results of this study showed a significant association between visual system impairment with macroglia and microglia activation in the MOG-induced EAE model. The novel oral immunomodulatory agent fingolimod could alleviate the immune response in the retina and optic nerve, which are the primary targets in MS. Considering the paradox of favorable and side effects of fingolimod on visual system, we speculate that side effects including macular oedema caused by fingolimod during MS treatment is tendency to be vasogenic rather than hypergliosis in optic nerve and retina which warrants further neuroophthalmological investigation.

## Data Availability Statement

The original contributions presented in the study are included in the article/supplementary material, further inquiries can be directed to the corresponding author/s.

## Ethics Statement

The animal study was reviewed and approved by the Ethics Committee of Capital Medical University (AEEI-2019-186).

## Author Contributions

TY drafted this manuscript and conceived of the study and participated in its design. TY, ZZ, XW, YK, YT, and XZ performed H&E, LFB, and IF. XY and TY performed the statistical analysis. TY, LW, and YF participated in the design and coordination of the study. All authors read and approved the final manuscript.

## Conflict of Interest

The authors declare that the research was conducted in the absence of any commercial or financial relationships that could be construed as a potential conflict of interest.

## References

[B1] Aktas O. Küry P. Kieseier B. Hartung H. P. (2010). Fingolimod is a potential novel therapy for multiple sclerosis. *Nat. Rev. Neurol.* 6 373–382. 10.1038/nrneurol.2010.76 20551946

[B2] Ambrosius B. Pitarokoili K. Schrewe L. Pedreiturria X. Motte J. Gold R. (2017). Fingolimod attenuates experimental autoimmune neuritis and contributes to Schwann cell-mediated axonal protection. *J. Neuroinflamm.* 14:92. 10.1186/s12974-017-0864-z 28446186 PMC5406994

[B3] Brambilla R. (2019). The contribution of astrocytes to the neuroinflammatory response in multiple sclerosis and experimental autoimmune encephalomyelitis. *Acta Neuropathol.* 137 757–783. 10.1007/s00401-019-01980-7 30847559 PMC6483860

[B4] Bravo B. Gallego M. I. Flores A. I. Bornstein R. Puente-Bedia A. Hernández J. (2016). Restrained Th17 response and myeloid cell infiltration into the central nervous system by human decidua-derived mesenchymal stem cells during experimental autoimmune encephalomyelitis. *Stem Cell Res. Ther.* 7:43. 10.1186/s13287-016-0304-5 26987803 PMC4797118

[B5] Bringmann A. Pannicke T. Grosche J. Francke M. Wiedemann P. Skatchkov S. N. (2006). Müller cells in the healthy and diseased retina. *Prog. Retin. Eye Res.* 25 397–424. 10.1016/j.preteyeres.2006.05.003 16839797

[B6] Chan J. W. (2002). Optic neuritis in multiple sclerosis. *Ocul. Immunol. Inflamm.* 10 161–186. 10.1076/ocii.10.3.161.15603 12789593

[B7] Chao C. C. Gutiérrez-Vázquez C. Rothhammer V. Mayo L. Wheeler M. A. Tjon E. C. (2019). Metabolic control of astrocyte pathogenic activity via cPLA2-MAVS. *Cell* 179 1483–1498.e22. 10.1016/j.cell.2019.11.016 31813625 PMC6936326

[B8] Chiba K. Kataoka H. Seki N. Shimano K. Koyama M. Fukunari A. (2011). Fingolimod (FTY720), sphingosine 1-phosphate receptor modulator, shows superior efficacy as compared with interferon-β in mouse experimental autoimmune encephalomyelitis. *Int. Immunopharmacol.* 11 366–372. 10.1016/j.intimp.2010.10.005 20955831

[B9] Chitnis T. Arnold D. L. Banwell B. Brück W. Ghezzi A. Giovannoni G. (2018). Trial of fingolimod versus interferon beta-1a in pediatric multiple sclerosis. *N. Engl. J. Med.* 379 1017–1027. 10.1056/NEJMoa1800149 30207920

[B10] Chu F. Shi M. Zheng C. Shen D. Zhu J. Zheng X. (2018). The roles of macrophages and microglia in multiple sclerosis and experimental autoimmune encephalomyelitis. *J. Neuroimmunol.* 318 1–7. 10.1016/j.jneuroim.2018.02.015 29606295

[B11] Cohen J. A. Barkhof F. Comi G. Hartung H. P. Khatri B. O. Montalban X. (2010). Oral fingolimod or intramuscular interferon for relapsing multiple sclerosis. *N. Engl. J. Med.* 362 402–415. 10.1056/NEJMoa0907839 20089954

[B12] Colombo E. Di Dario M. Capitolo E. Chaabane L. Newcombe J. Martino G. (2014). Fingolimod may support neuroprotection via blockade of astrocyte nitric oxide. *Ann. Neurol.* 76 325–337. 10.1002/ana.24217 25043204

[B13] Derfuss T. Mehling M. Papadopoulou A. Bar-Or A. Cohen J. A. Kappos L. (2020). Advances in oral immunomodulating therapies in relapsing multiple sclerosis. *Lancet Neurol* 19 336–347. 10.1016/S1474-4422(19)30391-6 32059809

[B14] Eken A. Duhen R. Singh A. K. Fry M. Buckner J. H. Kita M. (2017). S1P1 deletion differentially affects TH17 and regulatory T cells. *Sci. Rep.* 7:12905. 10.1038/s41598-017-13376-2 29018225 PMC5635040

[B15] Gelfand J. M. Nolan R. Schwartz D. M. Graves J. Green A. J. (2012). Microcystic macular oedema in multiple sclerosis is associated with disease severity. *Brain* 135 1786–1793. 10.1093/brain/aws098 22539259 PMC3359753

[B16] Giralt M. Ramos R. Quintana A. Ferrer B. Erta M. Castro-Freire M. (2013). Induction of atypical EAE mediated by transgenic production of IL-6 in astrocytes in the absence of systemic IL-6. *Glia* 61 587–600. 10.1002/glia.22457 23322593

[B17] Gonçalves F. F. Lucatto L. de Camargo A. S. Falcão A. B. Melo L. Jr. Andrade E. P. (2019). Retinal nerve fibre layer evaluation in multiple sclerosis patients with and without optic neuritis. *Acta Ophthalmol.* 97 e662–e663. 10.1111/aos.13947 30318736

[B18] Groves A. Kihara Y. Jonnalagadda D. Rivera R. Kennedy G. Mayford M. (2018). A Functionally defined in vivo astrocyte population identified by c-Fos activation in a mouse model of multiple sclerosis modulated by s1p signaling: immediate-early Astrocytes (ieAstrocytes). *eNeuro* 5:ENEURO.0239-18.2018. 10.1523/ENEURO.0239-18.2018 30255127 PMC6153337

[B19] Horstmann L. Kuehn S. Pedreiturria X. Haak K. Pfarrer C. Dick H. B. (2016). Microglia response in retina and optic nerve in chronic experimental autoimmune encephalomyelitis. *J. Neuroimmunol.* 298 32–41. 10.1016/j.jneuroim.2016.06.008 27609273

[B20] Hu P. Hunt N. H. Arfuso F. Shaw L. C. Uddin M. N. Zhu M. (2017). Increased Indoleamine 2,3-dioxygenase and quinolinic acid expression in microglia and müller cells of diabetic human and rodent retina. *Invest. Ophthalmol. Vis. Sci.* 58 5043–5055. 10.1167/iovs.17-21654 28980000 PMC5633007

[B21] Huwiler A. Zangemeister-Wittke U. (2018). The sphingosine 1-phosphate receptor modulator fingolimod as a therapeutic agent: recent findings and new perspectives. *Pharmacol. Ther.* 185 34–49. 10.1016/j.pharmthera.2017.11.001 29127024

[B22] Kappos L. O’Connor P. Radue E. W. Polman C. Hohlfeld R. Selmaj K. (2015). Long-term effects of fingolimod in multiple sclerosis: the randomized FREEDOMS extension trial. *Neurology* 84 1582–1591. 10.1212/WNL.0000000000001462 25795646 PMC4408283

[B23] Kappos L. Radue E. W. O’Connor P. Polman C. Hohlfeld R. Calabresi P. (2010). A placebo-controlled trial of oral fingolimod in relapsing multiple sclerosis. *N. Engl. J. Med.* 362 387–401. 10.1056/NEJMoa0909494 20089952

[B24] Karussis D. (2014). The diagnosis of multiple sclerosis and the various related demyelinating syndromes: a critical review. *J. Autoimmun.* 48–49 134–142. 10.1016/j.jaut.2014.01.022 24524923

[B25] Kataoka H. Sugahara K. Shimano K. Teshima K. Koyama M. Fukunari A. (2005). FTY720, sphingosine 1-phosphate receptor modulator, ameliorates experimental autoimmune encephalomyelitis by inhibition of T cell infiltration. *Cell. Mol. Immunol.* 2 439–448. 16426494

[B26] Kolappan M. Henderson A. P. Jenkins T. M. Wheeler-Kingshott C. A. Plant G. T. Thompson A. J. (2009). Assessing structure and function of the afferent visual pathway in multiple sclerosis and associated optic neuritis. *J. Neurol.* 256 305–319. 10.1007/s00415-009-0123-z 19296047

[B27] Liddelow S. A. Guttenplan K. A. Clarke L. E. Bennett F. C. Bohlen C. J. Schirmer L. (2017). Neurotoxic reactive astrocytes are induced by activated microglia. *Nature* 541 481–487. 10.1038/nature21029 28099414 PMC5404890

[B28] Lindenau W. Kuhrt H. Ulbricht E. Körner K. Bringmann A. Reichenbach A. (2019). Cone-to-Müller cell ratio in the mammalian retina: a survey of seven mammals with different lifestyle. *Exp. Eye Res.* 181 38–48. 10.1016/j.exer.2019.01.012 30641045

[B29] Mandal P. Gupta A. Fusi-Rubiano W. Keane P. A. Yang Y. (2017). Fingolimod: therapeutic mechanisms and ocular adverse effects. *Eye* 31 232–240. 10.1038/eye.2016.258 27886183 PMC5306460

[B30] Palmgren A. (1948). A rapid method for selective silver staining of nerve fibres and nerve endings in mounted paraffin sections. *Acta Zool.* 29 377–392. 10.1111/j.1463-6395.1948.tb00032.x

[B31] Ponomarev E. D. Veremeyko T. Barteneva N. Krichevsky A. M. Weiner H. L. (2011). MicroRNA-124 promotes microglia quiescence and suppresses EAE by deactivating macrophages via the C/EBP-α-PU.1 pathway. *Nat. Med.* 17 64–70. 10.1038/nm.2266 21131957 PMC3044940

[B32] Qiu X. Guo Q. Liu X. Luo H. Fan D. Deng Y. (2018). Pien Tze huang alleviates relapsing-remitting experimental autoimmune encephalomyelitis mice by regulating Th1 and Th17 cells. *Front. Pharmacol.* 9:1237. 10.3389/fphar.2018.01237 30429789 PMC6220046

[B33] Rossi S. Lo Giudice T. De Chiara V. Musella A. Studer V. Motta C. (2012). Oral fingolimod rescues the functional deficits of synapses in experimental autoimmune encephalomyelitis. *Br. J. Pharmacol.* 165 861–869. 10.1111/j.1476-5381.2011.01579.x 21740406 PMC3312484

[B34] Rothhammer V. Borucki D. M. Tjon E. C. Takenaka M. C. Chao C. C. Ardura-Fabregat A. (2018). Microglial control of astrocytes in response to microbial metabolites. *Nature* 557 724–728. 10.1038/s41586-018-0119-x 29769726 PMC6422159

[B35] Rothhammer V. Kenison J. E. Tjon E. Takenaka M. C. de Lima K. A. Borucki D. M. (2017). Sphingosine 1-phosphate receptor modulation suppresses pathogenic astrocyte activation and chronic progressive CNS inflammation. *Proc. Natl. Acad. Sci. U.S.A.* 114 2012–2017. 10.1073/pnas.1615413114 28167760 PMC5338419

[B36] Sospedra M. Martin R. (2016). Immunology of multiple sclerosis. *Semin. Neurol.* 36 115–127. 10.1055/s-0036-1579739 27116718

[B37] Tassoni A. Farkhondeh V. Itoh Y. Itoh N. Sofroniew M. V. Voskuhl R. R. (2019). The astrocyte transcriptome in EAE optic neuritis shows complement activation and reveals a sex difference in astrocytic C3 expression. *Sci. Rep.* 9:10010. 10.1038/s41598-019-46232-6 31292459 PMC6620300

[B38] Tsai H. C. Han M. H. (2016). Sphingosine-1-Phosphate (S1P) and S1P signaling pathway: therapeutic targets in autoimmunity and inflammation. *Drugs* 76 1067–1079. 10.1007/s40265-016-0603-2 27318702

[B39] Wang M. Wong W. T. (2014). Microglia-Müller cell interactions in the retina. *Adv. Exp. Med. Biol.* 801 333–338. 10.1007/978-1-4614-3209-8_42 24664715 PMC4685688

[B40] Wheeler M. A. Jaronen M. Covacu R. Zandee S. Scalisi G. Rothhammer V. (2019). Environmental control of astrocyte pathogenic activities in CNS inflammation. *Cell* 176 581–596.e18. 10.1016/j.cell.2018.12.012 30661753 PMC6440749

[B41] Yamasaki R. Lu H. Butovsky O. Ohno N. Rietsch A. M. Cialic R. (2014). Differential roles of microglia and monocytes in the inflamed central nervous system. *J. Exp. Med.* 211 1533–1549. 10.1084/jem.20132477 25002752 PMC4113947

[B42] Zarbin M. A. Jampol L. M. Jager R. D. Reder A. T. Francis G. Collins W. (2013). Ophthalmic evaluations in clinical studies of fingolimod (FTY720) in multiple sclerosis. *Ophthalmology* 120 1432–1439. 10.1016/j.ophtha.2012.12.040 23531349

[B43] Zhang C. J. Jiang M. Zhou H. Liu W. Wang C. Kang Z. (2018). TLR-stimulated IRAKM activates caspase-8 inflammasome in microglia and promotes neuroinflammation. *J. Clin. Invest.* 128 5399–5412. 10.1172/JCI121901 30372424 PMC6264724

[B44] Zhang J. Zhang Z. G. Li Y. Ding X. Shang X. Lu M. (2015). Fingolimod treatment promotes proliferation and differentiation of oligodendrocyte progenitor cells in mice with experimental autoimmune encephalomyelitis. *Neurobiol. Dis.* 76 57–66. 10.1016/j.nbd.2015.01.006 25680941

